# A Pipeline for Automated Quality Control of Chest Radiographs

**DOI:** 10.1148/ryai.240003

**Published:** 2025-03-05

**Authors:** Ian A. Selby, Eduardo González Solares, Anna Breger, Michael Roberts, Lorena Escudero Sánchez, Judith Babar, James H. F. Rudd, Nicholas A. Walton, Evis Sala, Carola-Bibiane Schönlieb, Jonathan R. Weir-McCall, for the AIX-COVNET Collaboration

**Affiliations:** ^1^Department of Radiology, University of Cambridge, 218 Cambridge Biomedical Campus, Cambridge CB2 0QQ, England; ^2^Department of Radiology, Cambridge University Hospitals National Health Services Trust, Cambridge Biomedical Campus, Cambridge, England; ^3^Institute of Astronomy, University of Cambridge, Cambridge, England; ^4^Department of Applied Mathematics and Theoretical Physics, University of Cambridge, Cambridge, England; ^5^Center of Medical Physics and Biomedical Engineering, Medical University of Vienna, Vienna, Austria; ^6^School of Clinical Medicine, University of Cambridge, Cambridge Biomedical Campus, Cambridge, England; ^7^Cancer Research UK Cambridge Centre, Cambridge Biomedical Campus, Cambridge, England; ^8^Department of Diagnostic Imaging and Oncological Radiotherapy, Policlinico Universitario A. Gemelli IRCCS, Rome, Italy; ^9^Department of Radiological and Hematological Sciences, Universita Cattolica del Sacro Cuore, Rome, Italy; ^10^Department of Radiology, Royal Papworth Hospital, Cambridge, England; ^11^Department of Cardiovascular Imaging, School of Biomedical Engineering and Imaging Sciences, King’s College London, London, England; ^12^Members of the AIX-COVNET Collaboration are listed at the end of this article.

**Keywords:** Artificial Intelligence, Automated Data Curation, Automated Quality Control, Thoracic Radiography, Data Standardization

## Abstract

This article presents a suite of quality control tools for chest radiographs based on traditional and artificial intelligence methods, developed and tested with data from 39 centers in seven countries.

See also commentary by Yanagawa and Sato in this issue.

*Supplemental material is available for this article.*

**Summary**

This article presents a suite of quality control tools for chest radiographs based on traditional and artificial intelligence methods, developed and tested with data from 39 centers in seven countries.

**Key Points**

■ An open-source, publicly available automated quality control tool was developed that can reproducibly and quickly curate large datasets of chest radiographs, allowing radiologists to concentrate on more complex development tasks.
■ The tool enhances the efficiency of expert quality control by providing standardized data extraction and preprocessing, statistical insights and visual summaries, and intelligently recommended subsets of data for targeted manual expert review or exclusion.
■ Two large multicenter datasets from the United Kingdom and Spain were used for development, with three other independent datasets reserved for model evaluation to ensure robust assessment of generalization across diverse patient populations, imaging equipment, and clinical settings.

## Introduction

Despite the potential of artificial intelligence to help analyze medical imaging, a systematic review performed 1 year after the onset of COVID-19 revealed a lack of clinically viable radiologic models (1). One major reason for this is shortcut learning, in which models learn unintended, nonpathologic predictors, such as patient positioning or text annotations, which are spuriously correlated with the outcome (Fig S1) (2–6). This phenomenon is not exclusive to COVID-19 (7) or certain model architectures, and adding additional data does not necessarily help (8). Collecting more accurately annotated, less biased data can reduce shortcut learning, enhancing the generalization of downstream artificial intelligence models (2). Comprehensive data analysis and quality control (QC) before model development are needed to identify potential issues, allowing developers to mitigate their impact through techniques such as stratification and adversarial training (2,8,9).

Using large, radiologist-annotated multinational datasets, we developed a comprehensive, freely available pipeline of classic statistical and artificial intelligence–based tools for automated QC and standardization of chest radiographs called AutoQC. This pipeline aims to automatically curate large datasets to give developers an overview and statistics, while suggesting a subset of data for targeted manual review. To ensure the accuracy of our pipeline across a wide range of radiographic equipment and datasets, we assess the performance of our tools on external datasets as compared with expert annotations.

## Materials and Methods

The Brent Research Ethics Committee provided ethical approval for this retrospective study (Integrated Research Application System: 282705, Research Ethics Committee: 20/Health Research Authority/2504, R&D: A095585). Informed consent was not required.

### Datasets

We used two large multicenter datasets from the United Kingdom and the Valencia region of Spain for development, with three other independent datasets reserved for model testing (Table 1). A single radiograph was randomly selected for any patient with multiple images to minimize patient-level bias.

**Table 1:**
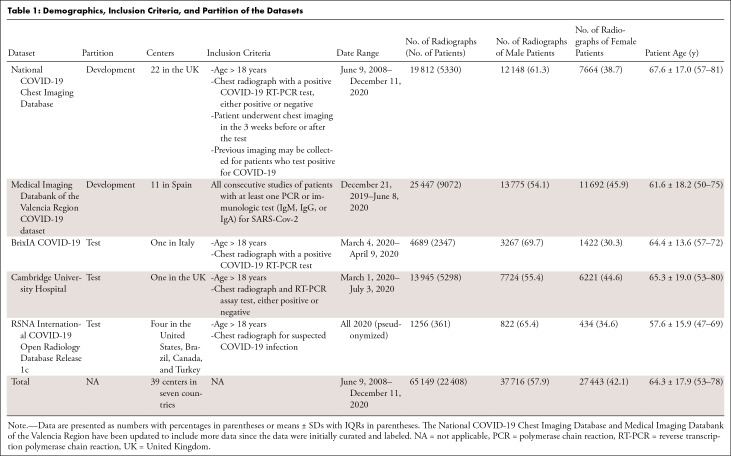
Demographics, Inclusion Criteria, and Partition of the Datasets

The training data were partitioned into training (64%), tuning (also known as *validation*, 16%), and test sets (20%). We used stratification to ensure consistent label proportions across partitions, which included known shortcuts and biases identified from the current literature: equipment manufacturer (2,7,10), deviation index (as a surrogate for exposure and contrast) (8,11), sex and gender (2,4,12,13), age (4,12–14), and ethnicity (12,15–17). When these data were missing, the patients were excluded. If an age range was provided, the midpoint was used.

### Data Annotation

**Figure 1:**
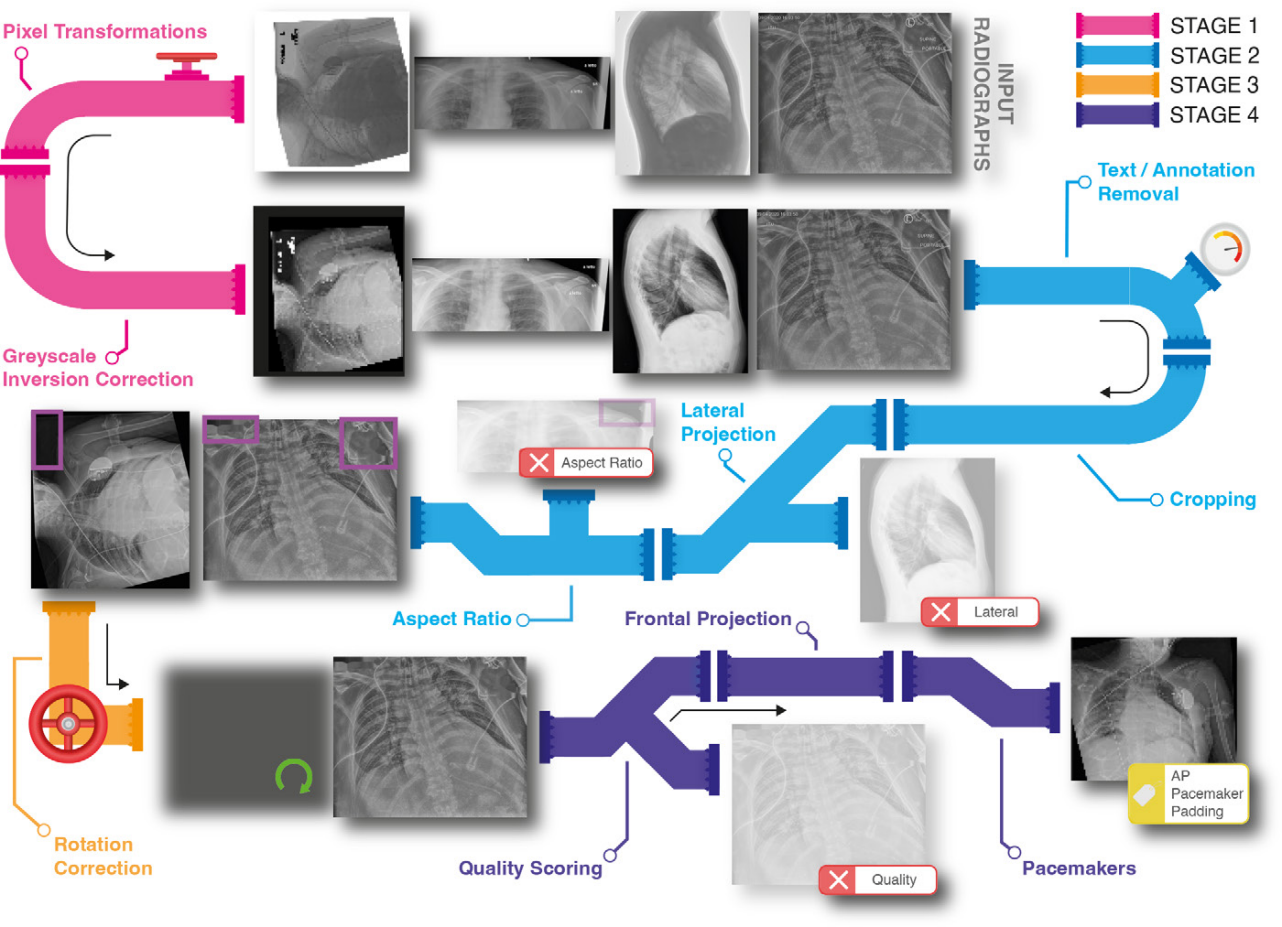
Graphic of the automated quality control (AutoQC) pipeline workflow with the four input radiographs at the top. Annotation removal is highlighted with purple boxes, with a green arrow showing an image after rotation correction. These four radiographs were selected for illustrative purposes, and the proportion rejected does not reflect the pipeline when deployed. AP = anteroposterior.

**Figure 2:**
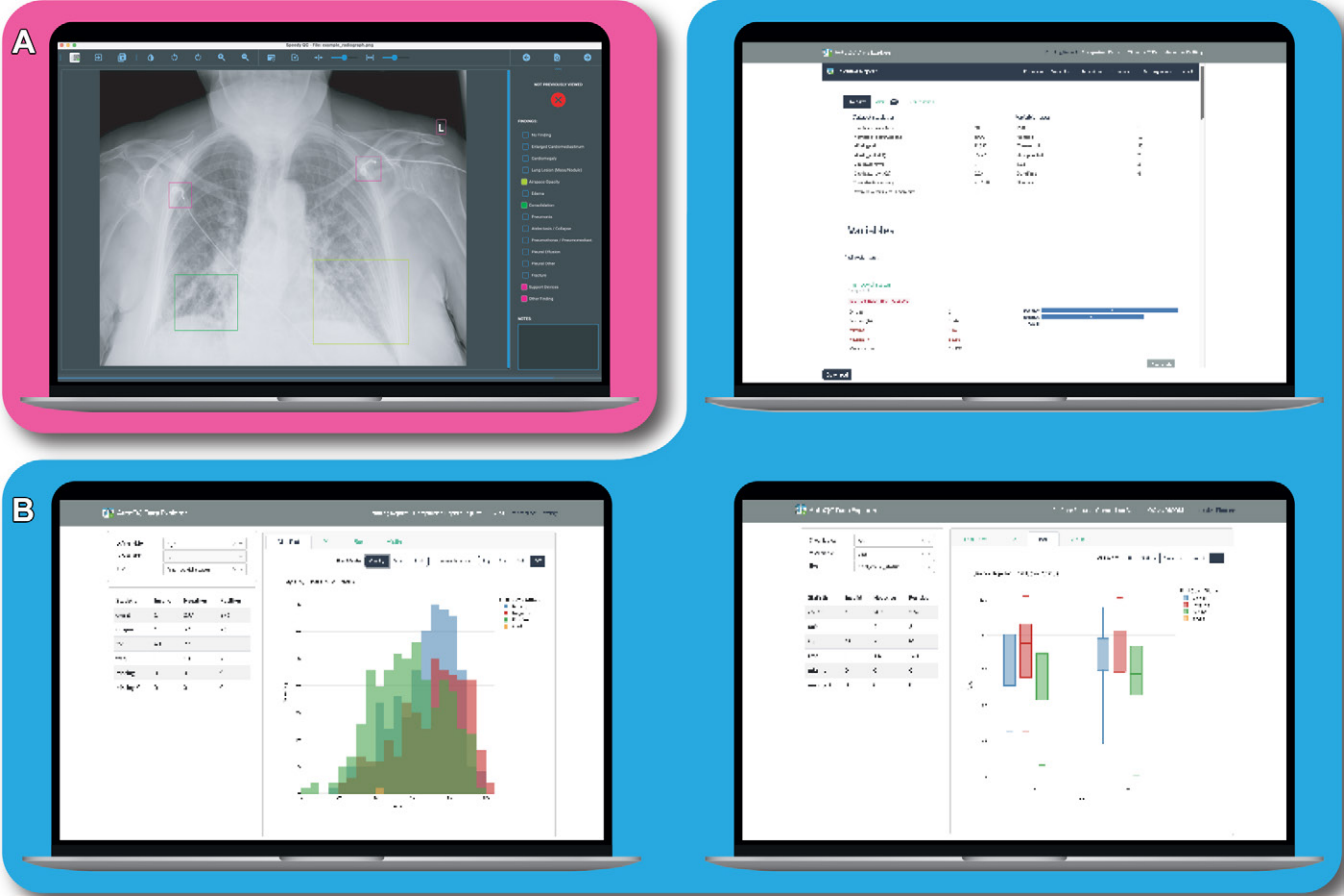
Screenshots of the SpeedyAnnotate annotation tool (**A**, pink) and the automated quality control (AutoQC) data explorer (**B**, blue). **(A)** SpeedyAnnotate is freely available online *(https://gitlab.developers.cam.ac.uk/maths/cia/covid-19-projects/speedyannotate)* and can be customized to use checkboxes and/or radio buttons, with the option to draw bounding boxes. The graphical user interface is built using the PyQt6 package. **(B)** The analysis package, built with Dash *(https://dash.plotly.com/)*, produces a report using the ydata profiling *(https://docs.profiling.ydata.ai)* of relevant metadata (top) and the quality control labels but also allows for interactive plotting of variables and comparison of the AutoQC outputs with Digital Imaging and Communications in Medicine metadata (bottom).

An interdisciplinary team of radiologists and data scientists identified potentially spurious correlations for inclusion in the AutoQC pipeline (Fig 1). Given the unambiguous nature of the task, radiographs were labeled for training and testing by a single radiologist in training (I.A.S., 4 years of chest radiography experience), who was supervised by an experienced cardiothoracic radiologist (J.B., 15 years of chest radiography experience). We used a custom graphic user interface to facilitate annotation *(https://gitlab.developers.cam.ac.uk/maths/cia/covid-19-projects/speedyannotate)* (Fig 2A).

### The QC Pipeline

AutoQC is organized into four stages, each standardizing an image ready for the next stage and marking undesirable radiographs for review or exclusion depending on the settings (Table 2; Figs 1, S2). Table 2 provides the exclusion criteria for the development at each stage. Appendixes S3–S6 give detailed methods for each tool, organized by stage in the pipeline.

**Table 2:**
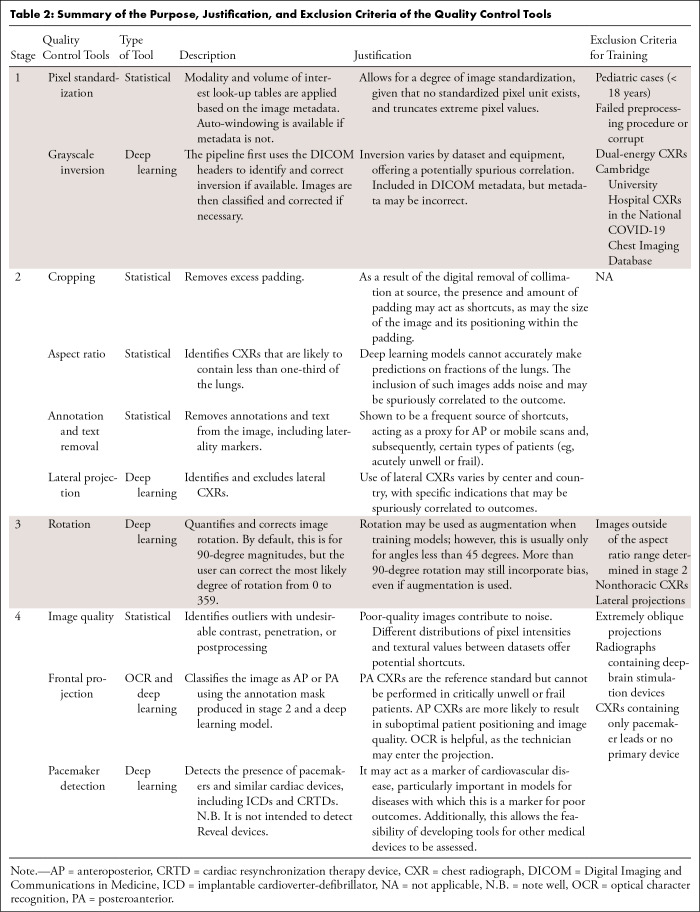
Summary of the Purpose, Justification, and Exclusion Criteria of the Quality Control Tools

After a user runs the pipeline, an interactive report is generated, providing statistics and graphic visualizations of the QC labels and the image metadata (Fig 2B). The AutoQC Python package and all code used are available on GitLab *(https://gitlab.developers.cam.ac.uk/maths/cia/covid-19-projects/autoqc)* with documentation *(https://maths.uniofcam.dev/cia/covid-19-projects/autoqc/)*. The multiplatform package can be run on a central processing unit, multiple central processing units, a graphics processing unit, or a server. To calculate runtimes, 250 radiographs were randomly selected from each test set and run through the pipeline.

### Model Evaluation

All tools were evaluated using the following metrics, calculated using bootstrapping of the test set (*n* = 1000): area under the receiver operating characteristic curve, accuracy, precision (also known as *positive predictive value*), recall (also known as *sensitivity* or *true-positive rate*), specificity, negative predictive value, and F1 score. The scikit-learn Python library (18) was used to sample the data and calculate the metrics.

### Evaluation of the Impact of Annotation and Text Removal on Classification of Radiographic Projection

To demonstrate the pipeline’s beneficial impact, we compared the results of frontal projection models trained on images with text removed using our tool, the original radiographs, and radiographs with text obscured using bounding boxes of zero intensity. Each model was evaluated on data with and without text removed and on a subset with no annotations either intrinsically or removed by manual cropping. To quantitatively analyze which model focused on areas of annotations the least, we calculated the mean intersection over union (also known as the Jaccard index) for the annotation masks and the gradient-weighted class activation maps for each model.

## Results

### Dataset Characteristics

Data from five repositories covering 39 centers across seven countries were used, including 65 149 radiographs from 22 408 patients. After applying inclusion criteria and randomly selecting one image for each patient, 21 862 radiographs were included in the study.

### Model Performance

The metrics for evaluating the tools on external test sets are summarized in Table 3, with the corresponding confusion matrices presented in Figure S7. The most crucial results are captured in Table 3, and detailed analyses for each tool are provided in Appendixes S3–S6. Additional significant findings not featured in Table 3 are summarized as follows:

**Table 3:**
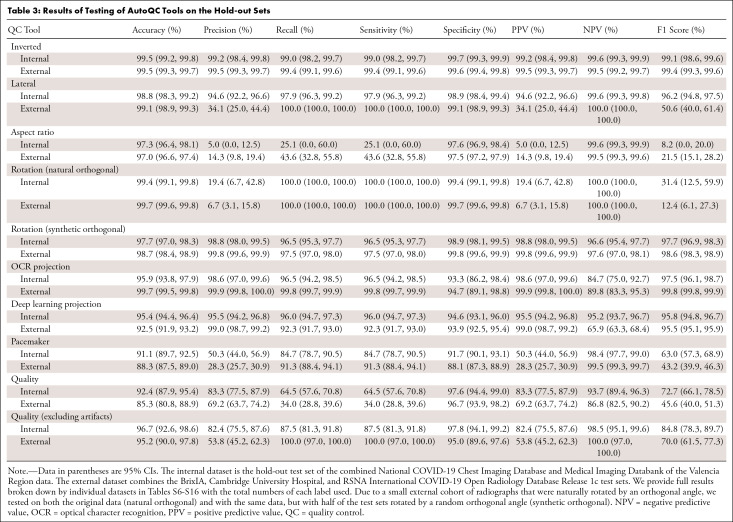
Results of Testing of AutoQC Tools on the Hold-out Sets

***Pixel intensity transformations.***

The coefficient of variation, mean SD, and mean entropy of the radiographs all decreased after look-up table application. The standardized mean difference was reduced for all metrics between different manufacturers (Appendix S3).

***Annotation and text removal.***

The tool entirely removed annotations from 88.5% (95% CI: 86.6, 90.4), with 8.9% (95% CI: 7.2, 10.6) having none to remove. The tool partially removed annotations for 2.3% (95% CI: 1.4, 3.2) and failed to remove any for 0.4% (95% CI: 0, 0.8). A total of 0.47% (95% CI: 0.06, 0.89) experienced inappropriate inpainting within the lungs due to jewelry or metallic clothing artifacts (Appendix S4).

***Cropping.***

Most radiographs underwent cropping (57.2%; 4319 of 7556; Fig S34), with no inappropriate cropping identified (Appendix S4).

***Aspect ratio.***

The optimal bounds were 0.85 and 1.48 for the development set (Appendix S4).

***Quality scoring.***

The sensitivity and specificity for a poor-quality image were 34.0% (95% CI: 28.8, 39.6) and 96.7% (95% CI: 93.9, 98.2), respectively. The low sensitivity resulted from a failure to identify imperfections such as scratches or pixel drop-out, particularly prevalent in the RSNA International COVID-19 Open Radiology Database Release 1c dataset. When images with these artifacts were excluded, the sensitivity increased to 100% (95% CI: 97.0, 100.0), successfully identifying radiographs with excessively high sharpness, low contrast, blurring, and large collimation or residual padding areas.

### Inconsistencies in Digital Imaging and Communications in Medicine Metadata

While evaluating the tools, we found Digital Imaging and Communications in Medicine metadata unreliable in several areas. For example, the Series Description header contained a projection in only 56.5% (12 580 of 22 274) and would have incorrectly labeled a lateral radiograph as a frontal projection in 2.9% (364 of 12 580).

### Additional Results and Runtimes

Additional results tables and charts are also provided: the patient demographics within the partitions (Table S1), flowchart of studies (Fig S6), model and tuner hyperparameters (Table S4), model thresholds (Table S5), results by external test set (Tables S6–S15), receiver operating characteristic and calibration curves with confusion matrices for each test set (Figs S9–S16), and examples of gradient-weighted class activation maps (Figs S17–S21) and incorrect classifications (Figs S22–S28). Figures S44–S54 contain the image quality assessment outputs.

The final pipeline took approximately 17 seconds per image to process Digital Imaging and Communications in Medicine–format radiographs using 100 central processing units and 1 graphics processing unit (runtime results are in Table S3).

### Effect of Text and Annotation Removal on Projection Classification

When comparing models trained to detect frontal projection with and without text removal, the model trained using the original radiographs (without text removed) showed a significant decrease in prediction accuracy, sensitivity, and F1 score when tested on radiographs where the text had been removed. However, models trained using images without text performed well regardless of whether text removal techniques such as inpainting or bounding boxes were used. There was a statistically significant difference in the mean intersection-over-union scores, with the lowest indicating the least reliance on text removal and inpainting to identify projection when annotations were removed using inpainting (Table S17). The complete results of testing the deep learning projection models trained with and without text removal are shown in Figure S8 and Table S16, and a more comprehensive write-up is provided in Appendix S7.

## Discussion

Our generalizable, automated pipeline can automatically curate, standardize, and provide a statistical overview of large chest radiograph datasets. It can highlight and address potential shortcuts in chest radiographs, suggesting a subset of images for a targeted review.

Radiologists must remain integral to the QC process, but improving its efficiency allows them to concentrate on more complex tasks. Furthermore, with Digital Imaging and Communications in Medicine data often incomplete or inaccurate, developers can improve performance of their models with confidence that spurious correlations have been minimized. The straightforward tool can be used with the out-of-the-box, fully trained models provided. It is freely available online to improve reproducibility, explainability, and fairness in radiographic data science. A full discussion of each tool is included in Appendixes S3–S6.

This study had limitations. The deep learning models may be susceptible to confounding, despite efforts to avoid this. The data were collected during the COVID-19 pandemic, which may reduce performance when applied to the current population. To ensure generalizability, testing on additional external datasets, such as MIMIC-CXR (19) or Chest X-ray 14 (20), is recommended, with tools being fine-tuned if performance declines. The study’s limitations are discussed in detail in Appendix S8.

Additional work to further demonstrate the benefit of such automated QC tools when deployed is planned, along with continued efforts to reduce shortcut learning in artificial intelligence for chest radiographs.

## Notes

**Keywords:** Artificial Intelligence, Automated Data Curation, Automated Quality Control, Thoracic Radiography, Data Standardization
